# High carbohydrate intakes may predict more inflammatory status than high fat intakes in pre-menopause women with overweight or obesity: a cross-sectional study

**DOI:** 10.1186/s13104-021-05699-1

**Published:** 2021-07-21

**Authors:** Elmira Karimi, Habib Yarizadeh, Leila Setayesh, Seyyedeh Forough Sajjadi, Nasim Ghodoosi, Leil Khorraminezhad, Khadijeh Mirzaei

**Affiliations:** 1 Department of Community Nutrition, School of Nutritional Sciences and Dietetics, Tehran University of Medical Sciences (TUMS), P.O. Box: 14155-6117, Tehran, Iran; 2 Endocrinology and Nephrology Unit, CHU de Québec-Laval University, Research Center, Québec, QC Canada

**Keywords:** Dietary fat, Dietary carbohydrate, Inflammation, Pre-menopause, Obesity

## Abstract

**Objective:**

The associations between dietary carbohydrate, fat intake, and inflammation are controversial. Most existing data are from industrialized societies which low-carbohydrate and high-fat diet is common and so their attribution to other populations remains unclear. We evaluated the association of fat and carbohydrate intakes with inflammatory markers in pre-menopause women with overweight or obesity in Iran.

**Results:**

Three hundred and sixty women with body mass index (BMI) ≥ 25 were included to this study. The levels of monocyte chemoattractant protein-1 (MCP-1) indicated a trend towards significance across tertiles of total dietary carbohydrate. We found that the levels of galectin-3 were negatively associated with dietary carbohydrate in adjusted model. In addition, the levels of MCP-1 and transforming growth factor beta (TGF-β) were positively correlated to dietary carbohydrate. No significant relationship was demonstrated between inflammatory parameters and total fat intake). However, there was a borderline significant negative association between total fat intake and TGF-β level in adjusted model. Therefore, a total dietary carbohydrate were related to elevated inflammation risk, while a total fat intake were not associated to higher inflammation. This study suggests reconsideration of applying global dietary guidelines in societies with high carbohydrate diet.

**Supplementary Information:**

The online version contains supplementary material available at 10.1186/s13104-021-05699-1.

## Introduction

The epidemic of cardiovascular diseases (CVDs) are continuing to increase an alarming rate in low and middle income societies [1]. Chronic inflammatory states which are identified with high levels of pro-inflammatory risk markers are involved in the pathogenesis of all stages of CVDs [2–4]. It has been shown that women with obesity have a higher probability of developing cardiovascular disorders than women with normal weight [5]. One of the important modifiable factor that contributes to chronic inflammatory state is diet [6].

Current dietary guidelines mostly have focused on lowering dietary total fat to < 30% of total energy intake [7], while higher carbohydrate intake play an important role to incidence chronic non-communicable diseases, such as CVDs [8]. Recommendations on restricting total fat intake are largely based on observational studies performed in European and North American societies where there is relatively high intakes of energy, total fats and low intake of total carbohydrate [9]. It remains unclear whether these guidelines can be applied in low and middle income countries where under-nutrition and high carbohydrate diet is significantly more common. Furthermore, these dietary guidelines are predominantly based on the assumption of a positive relationship between total fat intake and low-density lipoprotein cholesterol (LDL), and the association between LDL and CVDs events [10, 11]. There is an undeniable importance of taking inflammatory biomarkers from human investigations into account when recommendations on fat and carbohydrate intakes are discussed [12].

Despite the existence of several studies on the association between fat and carbohydrate intake with inflammation which are in line with the guidelines recommendations [13–17], recently, a number of studies did not support the previous results. For instance, the recent meta-analyses indicated either no or inverse association between fat intake with inflammatory biomarkers, risk of CVDs, and mortality [18–20]. Moreover, findings from several studies did not support the anti-inflammatory benefits of high consumption of carbohydrates [21–23]. The uncertainty regarding the association between consumption of fat and carbohydrate with inflammatory markers might be attributed to differences in sex, ethnicity, use of the different food frequency questionnaires (FFQ) as well as the fact that most of these studies have been performed in European and North American societies. This study provided a unique opportunity to assess the association between fat and carbohydrate intake amounts with inflammatory factors in Iran where under-nutrition and high-carbohydrate diet is of greater concern.

## Main text

### Materials and methods

#### Study sample and design

Current study hypothesized that in societies with high carbohydrate diet, the association between total carbohydrate and inflammation might be stronger than the relationship between dietary fat and inflammation. This observational investigation was a multi-center cross-sectional study that was performed by a multistage cluster random sampling method. A number of 360 women who referred to community health centers of Tehran University of Medical Science were recruited for this investigation. The protocol for the recruitment of participants was described in the previous papers by our team [24, 25]. Inclusion criteria were consisted of healthy women aged 18–50 with BMI equal or more than 25. Exclusion criteria were those who had medical history of hypertension, addiction to alcohol, drugs and/or smoking, thyroid diseases, diabetes mellitus, CVDs, malignancies, hepatic or renal diseases, lactation, pregnancy and acute or chronic infections. This research was conducted according to the Declaration of Helsinki [26]. The protocol of this study was approved by the Ethics Committee of Tehran University of Medical Sciences, Tehran, Iran (With ID number: IR.TUMS.VCR.REC.1395.1234). Written and informed consent requirement was provided by all participants. Assessment of anthropometrics and biochemical variables, dietary intakes, physical activity and statistical analysis are provided in Additional file 1.

### Results

#### Research participants

Additional file 1: Table S1 presents the baseline characteristics, biochemical parameters for 360 pre-menopause women with overweight and obesity. The mean age, height, weight, and BMI of the study participants were 36.52 ± 8.32 years, 161.38 ± 5.70 cm, 78.97 ± 10.76 kg, and 30.33 ± 3.65 kg/m^2^, respectively. The mean of total carbohydrate and total fat intake in the study participants were 360.62 ± 114.79 g, range: 112.42–722.31) and 89.30 ± 32.75 g, range: 21.15–211.66), respectively. Among the participants, the frequency of low, moderate and high physical activity was 163, 117 and 10, respectively.

#### Primary findings

The general characteristics and inflammatory markers in study participants among tertiles of total dietary carbohydrate and fat intakes are shown in Additional file 1: Tables S2 and S3. Significant differences were found for age, body weight, and height across tertiles of dietary carbohydrate (P = 0.01, P = 0.02 and P = 0.01, respectively). Moreover, the levels of MCP-1 indicated an increasing trend towards significance among tertiles of dietary carbohydrate (P = 0.048).

#### Additional findings

Tables 1 and 2 indicate multiple linear regression analysis between inflammatory variables with dietary carbohydrate and fat, respectively. Furthermore, the levels of galectin-3 were independently and negatively associated with dietary carbohydrate in adjusted model (adjusted for energy intake, physical activity, and age) (P = 0.04) (Table 1). In addition, the levels of MCP-1 and TGF-β were independently and positively correlated to dietary carbohydrate in crude and adjusted models (P = 0.002 and P = 0.002 for MCP-1 and P = 0.03 and P = 0.003 for TGF-β for crude and adjusted models, respectively). As indicated in Table 2, there was borderline significant relationship between dietary fat intake and TGF-β in adjusted model (P = 0.05). However, no significant relationship was indicated between inflammatory parameters and dietary fat (P > 0.05).

**Table 1:** Assessment of the association between dietary carbohydrate intake and inflammatory markers

Inflammatory biomarkers	Model	Beta	CI 95%	P value
CRP (mg/L)	Model 1	0.00	− 0.37 to 0.37	0.96
	Model 2	0.05	− 0.26 to 0.58	0.45
IL-1β (mg/L)	Model 1	− 0.09	− 0.18 to 0.07	0.40
	Model 2	− 0.09	− 0.21 to 0.11	0.51
TGF-β (mg/L)	Model 1	0.57	5.63 to 104.45	**0.03**
	Model 2	1.23	52.71 to 184.40	**0.003**
Galectin-3 (mg/L)	Model 1	− 0.19	− 1.84 to 0.13	0.09
	Model 2	− 0.28	− 2.43 to − 0.01	**0.04**
MCP-1 (mg/L)	Model 1	0.23	2.53 to 11.38	**0.002**
	Model 2	0.29	3.28 to 14.02	**0.002**

Multiple linear regression analysis

*CRP* C-reactive protein, *IL1β* interleukin 1 beta, *TGF-β* transforming growth factor beta, *MCP-1* monocyte chemoattractant protein-1, *Model 1* Crude model, *Model 2* adjusted model for age, *BMI* physical activity and calorie intake

**Table 2:** Assessment of the association between dietary fat intake and inflammatory markers

Inflammatory biomarkers	Model	Beta	CI 95%	P value
CRP (mg/L)	Model 1	0.00	− 0.26 to 0.29	0.91
	Model 2	0.02	− 0.29 to 0.39	0.79
IL-1β (mg/L)	Model 1	0.13	− 0.04 to 0.16	0.26
	Model 2	0.14	− 0.07 to 0.20	0.35
TGF-β (mg/L)	Model 1	− 0.19	− 40.25 to 20.85	0.50
	Model 2	− 0.95	− 95.06 to 0.15	**0.05**
Galectin-3 (mg/L)	Model 1	− 0.05	− 0.97 to 0.60	0.64
	Model 2	− 0.01	− 1.18 to 1.11	0.95
MCP-1 (mg/L)	Model 1	− 0.06	− 4.86 to 2.15	0.44
	Model 2	− 0.17	− 0.26 to 0.29	0.09

Multiple linear regression analysis. *CRP* C-reactive protein, *IL1β* interleukin 1 beta, *TGF-β* transforming growth factor beta, *MCP-1* monocyte chemoattractant protein-1, *Model 1* crude model, *Model 2* adjusted model for age, *BMI* physical activity and calorie intake

### Discussion

Current study aimed to investigate the association between intake of total carbohydrate and total fat with inflammatory markers in pre-menopausal women with overweight and obesity. Our findings illustrated that high-carbohydrate intake was associated with a high level of inflammatory state. While, fat intake did not have any statistically significant relationship with inflammatory markers. In fact, serum MCP-1 level was positively associated with total dietary carbohydrate. The TGF-β level had significant positive association with dietary carbohydrate amount. Moreover, the serum level of galectin-3 had significant negative association with dietary carbohydrate amount in adjusted model. An inverse correlation was also demonstrated between MCP-1 and TGF- β with fat amount in the diet, however this finding was insignificant, possibly due to small sample size.

For several years, dietary guidelines have recommended to decrease total fat intake and replacing them with carbohydrate which was based on the presumption of the association between high fat consumption and high low density lipoprotein level to inhibit CVDs events [10, 11].

The role of high inflammatory markers level in the risk of CVDs risk cannot be denied [12]. Currently, there is not a certain conclusion on the association between total fat and carbohydrate intake with inflammatory state which is probably because of the sensitivity of this association to various factors including ethnics, sociodemographic factors, types of the fat and carbohydrate consumed, age, and disease history [13, 14, 18, 21].

Present study was conducted among healthy pre-menopause women with obesity from a low-income country (an especial ethnic, disease background and gender). The consumption of a high-carbohydrate diet in a broad range (56.01–74% of total energy) which was predominantly from refined sources might explain the stronger correlation between carbohydrate intake and inflammatory state in this investigation than the previous ones, which were mainly performed on subjects in European and North American countries who have lower carbohydrate intake in a restricted range (35–56% of total energy) [27]. The current results were not in agreement with the guidelines. Based on the current findings, carbohydrate intake was positively associated with inflammatory state and therefore, people with high carbohydrate diet might benefit from reducing carbohydrate intake and elevating the intake of fats. Moreover, this study used several inflammatory and anti-inflammatory (galectin-3) factors which are less assessed in the previous studies on this subject.

Current study suggested that high-carbohydrate diet can probably led to an increase in the levels of MCP-1, a chemokine that the pro-inflammatory and adverse effects has been demonstrated in the several studies [28]. There might be several causes for less statistically significant linear association between total carbohydrate intake and MCP-1 levels such as an increasing effect of obesity on MCP-1 level and random errors in macronutrients intake calculation. The results of this investigation were in accordance with Forsythe et al.’s study [29] in which a very low carbohydrate diet led to a decrease in inflammatory markers such as tumor necrosis factor-alpha, MCP-1, interleukin-6, interleukin-8, and plasminogen activator inhibitor-1 compared to a low-fat diet. They expressed that the anti-inflammatory influences of carbohydrate restriction may be mediated through down-regulation of nuclear factor kappa B pathway [29, 30]. It has also been established that high-carbohydrate intake induces the production of the major lipogenic products and increases lipogenesis that it was related to higher levels of adiposity [29] which was associated with elevated levels of MCP-1 [28]. However, Hall et al. [31] demonstrated that the serum levels of MCP-1 remained constant after reduction of both fat and carbohydrate intake for 6-week among obese individuals. The inconsistency between results of current study and Hall et al. study might be due to various study designs and different sample sizes.

We found significant positive association between TGF-β level and dietary carbohydrate amount in this study. TGF-β is considered as a cytokine that regulates insulin resistance in obesity. In addition, it has been illustrated to induce macrophages proliferation and deposition in adipose tissue of obese mice [32]. Over-expression of TGF-β along with high-carbohydrate consumption was associated to high blood glucose level that led to stimulated IκB kinase phosphorylation and secretion of pro-inflammatory factors such as TGF-β via nuclear factor kappa B pathway [33, 34]. A long with the current results an experimental study indicated that high-carbohydrate diet, increased TGF-β, TNF-α and IL 1β, nuclear factor kappa light chain enhancer of activated B cells (NF-κB), and IL-6 in an animal study [34]. A human study also reported that low-carbohydrate diet with high amounts of fat contributed to reduction in serum level of TGF-β [35]. Near to significant negative correlation between total dietary fat and TGF-β was also indicated in present study which might have reached to significant level with a larger study population. There is no clear mechanism for this negative association. However, the insignificant reduction of body weight during dietary fat tertiles might have mediated the reduction of TGF-β in higher-fat intake [36]. Contrary to our results, Ohtomo et al. investigated the effects of high-carbohydrate/low-fat diet and middle-carbohydrate/middle-fat diet (as normal group) on hypertensive, obese, type 2 diabetic rats for 12-week which indicated that that the TGF-β in the kidney tissue reduced in the interventional diet compared to the control group. Inconsistency between human and animal findings might be due to unsuitability of attributing the results of animal models to humans [37].

There was negative association between serum galectin-3 level and dietary carbohydrate amount in adjusted model. Galectin-3 plays various and sometimes contradictory roles in pathological and physiological pathways depending on type of involved organs [38]. Several studies revealed that galectin-3 correlates with the prevention of chronic inflammatory diseases [38]. No study was presented on the association between macronutrients intakes with galectin-3 levels. Although, in line with current study, a clinical trial in mice with progressive hepatopathy indicated that low-carbohydrate and high-fat led to significant expression of galectin-3 gene in comparison to control diet which was probably due to improved mitochondria-related functions [39].

### Conclusion

In this study, dietary carbohydrate intake was associated with elevated MCP-1, TGF-β and reduced galectin-3 level which have been shown to predict the increase risk of CVDs disorders. Interestingly, this study did not find any association between total fat intake and pro-inflammatory markers.

This study indicated that high-carbohydrate diet predicted more inflammatory status than high-fats diets in Iran, which refined-carbohydrate are more consumed. The current results should be interpreted with caution and cannot be attributed to other populations. Further investigations on the association between intake of fat and carbohydrate with various pro- and anti-inflammatory factors and inflammatory diseases in various populations with larger sample sizes would be of interest.

### Limitations

The limitations of our article were small sample size and its cross-sectional design with no exact cause-effect and failure to follow the subjects during change in diet.

## Supplementary Information

**Additional file 1**:** Table S1. **The characteristics of demographic, anthropometric and laboratory parameters among study subjects. **Table S2. **The general characteristics and inflammatory parameters of participants across tertiles of dietary carbohydrate intake. **Table S3. **The general characteristics and inflammatory parameters of participants across tertiles of dietary fat intake. Assessment of anthropometrics and biochemical variables, dietary intakes, physical activity and statistical analysis are provided in Additional file.

## Data Availability

Not applicable.

## Abbreviations

BMI: Body mass index
MCP-1: Monocyte chemoattractant protein-1
TGF-β: Transforming growth factor beta
CVDs: Cardiovascular diseases
LDL: Low-density lipoprotein cholesterol
HDL: High-density lipoprotein cholesterol
FFQ: Food frequency questionnaires
WC: Waist circumference
WHR: Waist to hip ratio
IPAQ: International Physical Activity Questionnaire
ELISA: Enzyme-linked immunosorbent assay
