# Supplementary material for: High carbohydrate intakes may predict more inflammatory status than high fat intakes in pre-menopause women with overweight or obesity: a cross-sectional study
Source: BMC Res Notes. 2021 Jul 21;14:279. doi: 10.1186/s13104-021-05699-1 (PMC8296741; doi:10.1186/s13104-021-05699-1)
Supplement: Supplementary file 1 — Additional file 1: Table S1. The characteristics of demographic, anthropometric and laboratory parameters among study subjects. Table S2. The general characteristics and inflammatory parameters of participants across tertiles of dietary carbohydrate intake. Table S3. The general characteristics and inflammatory parameters of participants across tertiles of dietary fat intake. Assessment of anthropometrics and biochemical variables, dietary intakes, physical activity and statistical analysis are provided in Additional file. [file 13104_2021_5699_MOESM1_ESM.docx]

##### **Additional File 1**

##### **Assessment of anthropometrics variables, dietary intakes and physical activity**

##### An expert nutritionist measured the weight and the height of the subjects to the nearest 100 grams and 0.5 cm, respectively, using calibrated, digital scale (SECA, Vogel & Halke, Hamburg, Germany). By using this device, the procedure of standing and grabing the metal handles of the device were performed according to the manufacturer's instructions [1]. Waist circumference (WC) and hip circumference were also measured to the nearest 1.0 cm using an un-stretchable measuring tape. Waist to hip ratio (WHR) calculation was by dividing WC by hip circumference. The accuracy and validity of this study methods for the measurement of weight, height [2, 3], waist, and hip circumference [4] have been previously confirmed. BMI was calculated as body weight (kg) divided by square of the height (m^2^) and expressed as kg/m2. BMI ≥25 and ≥30 were interpreted as being overweight and obese, respectively [5]. Physical activity evaluation was done through the short form of the International Physical Activity Questionnaire (IPAQ) (Occupational, Transport, Yard/Garden, Household, and Leisure). IPAQ considered physical activity metabolic equivalent hours per week (MET-h/wk). The final added scores <600, 600-3500 and >3500 (MET-h/wk) were interpreted as low, moderate and strict physical activity, respectively [6]. Afterwards, general characteristics (age, smoking and alcohol dependency and medical history) were questioned from eligible participants. Usual dietary intake of subjects was obtained using 147-item semi-quantitative FFQ [7]. Trained dietitians, asked the participants to report their intake frequency for each food or drink item consumed over the past year in terms of day, week, month, and year. The reported frequency for each item was turned into a daily intake. The reported portion sizes of foods were then converted into grams per day by using household measures [8]. The reproducibility and relative validity of the 147-item FFQ were examined previously in the Tehran Lipid and Glucose Study [7].

##### The amount of total carbohydrate, total fat, and total energy for each food item was computed via Nutritionist IV software (version 7.0; NSquared Computing, Salem, OR, USA). However, for the traditional Iranian food item, such as kashk (Iranian dairy product) and traditional breads that do not exist in Nutritionist 4, the Iranian food composition table was applied [9].

##### The methods for the measurement of anthropometric indices and physical activity have also been published in our previous articles [1, 10].

##### **Clinical measurements**

##### The procedures of the biochemical measurement have been published in our previous studies [10-12]. All measurements were performed at the the Endocrinology & Metabolism Research Institute in Bionanotechnology laboratory of Tehran University of Medical Science. In summary, following a 12-h overnight fast, we collected venous blood samples from all study participants. The blood samples were centrifuged at 1000 x g for 15 min at 4°C. Then, plasma was immediately aliquoted into separate tubes and stored at −80°C. The extent of C-reactive protein was measured by use of an immunoturbidimetric assay (high-sensitivity assay by Hitachi 902). Total cholesterol, triglyceride, high-density lipoprotein cholesterol (HDL) and LDL were examined by using of enzymatic approaches and related kits (Pars Azemun, Iran) and auto analyzer system.  Furthermore, enzyme-linked immunosorbent assay (ELISA) method was used for the measurement of TGF-β (HUMAN TGF-BETA) and interleukin 1 beta (Quantikine ELIZA kit R&D System- USA). Galectin-3 was measured by Human Galactin-3*96 T, ELIZA kit Crystal Company. The ELISA method with an appropriate kit (Zell Bio GmbH, ULM, Germany was used for measuring MCP-1 levels.

##### **Statistical analysis**

##### The minimum sample size (equal to 347) was calculated through the following formula, in which r = 0.21, β = 0.95, and α = 0.05.

##### N= (([(Z1−α+Z1−β) ×√1−r^2^]/r)^2^+2),

However, due to data availability, we conducted study analysis on 360 participants. All statistical analyses were performed by “Statistical Package for the Social Sciences” (version 16.0; SPSS Inc, Chicago) and a P-value < 0.05 was considered as statistical significance. Normality testing of study variables was via Kolmogorov- Smirnov's analysis. Abnormal data were normalized following square or logarithmic transformations. Comparison of continues variables for each study member in tertiles of total fat and carbohydrate intakes was performed by one-way analysis of variance. In addition, multiple linear regression models (Crude and adjusted for age, BMI, physical activity and calorie intake) were used to assess the association of inflammatory variables with fat and carbohydrate consumptions.

### References

1. Mirzababaei A, Sajjadi SF, Ghodoosi N, Pooyan S, Arghavani H, Yekaninejad MS, Mirzaei K: **Relations of major dietary patterns and metabolically unhealthy overweight/obesity phenotypes among Iranian women.** *Diabetes Metab Syndr* 2019, **13:**322-331.

2. Kumar SN, Omar B, Htwe O, Joseph LH, Krishnan J, Jafarzedah Esfehani A, Min LL: **Reliability, agreement, and validity of digital weighing scale with MatScan in limb load measurement.** *J Rehabil Res Dev* 2014, **51:**591-598.

3. Geeta A, Jamaiyah H, Safiza MN, Khor GL, Kee CC, Ahmad AZ, Suzana S, Rahmah R, Faudzi A: **Reliability, technical error of measurements and validity of instruments for nutritional status assessment of adults in Malaysia.** *Singapore Med J* 2009, **50:**1013-1018.

4. Jaeschke L, Steinbrecher A, Pischon T: **Measurement of waist and hip circumference with a body surface scanner: feasibility, validity, reliability, and correlations with markers of the metabolic syndrome.** *PloS one* 2015, **10:**e0119430.

5. Weir CB, Jan A: **BMI classification percentile and cut off points.** *StatPearls [Internet]* 2019.

6. Hagströmer M, Oja P, Sjöström M: **The International Physical Activity Questionnaire (IPAQ): a study of concurrent and construct validity.** *Public Health Nutrition* 2006, **9:**755 - 762.

7. Mirmiran P, Esfahani FH, Mehrabi Y, Hedayati M, Azizi F: **Reliability and relative validity of an FFQ for nutrients in the Tehran lipid and glucose study.** *Public health nutrition* 2010, **13:**654-662.

8. Ghaffarpour M, Houshiar-Rad A, Kianfar H: **The manual for household measures, cooking yields factors and edible portion of foods.** *Tehran: Nashre Olume Keshavarzy* 1999, **7:**42-58.

9. Azar M, Sarkisian E: **Food composition table of Iran: National Nutrition and food research institute.** *Shaheed Beheshti University, Tehran* 1980.

10. Rasaei N, Kashavarz SA, Yekaninejad MS, Mirzaei K: **The association between sarcopenic obesity (SO) and major dietary patterns in overweight and obese adult women.** *Diabetes Metab Syndr* 2019, **13:**2519-2524.

11. Tavakoli A, Mirzababaei A, Sajadi F, Mirzaei K: **Circulating inflammatory markers may mediate the relationship between low carbohydrate diet and circadian rhythm in overweight and obese women.** *BMC Women's Health* 2021, **21:**1-10.

12. Ghodoosi N, Mirzababaei A, Rashidbeygi E, Badrooj N, Sajjadi SF, Setayesh L, Yekaninejad MS, Keshavarz SA, Shiraseb F, Mirzaei K: **Associations of dietary inflammatory index, serum levels of MCP-1 and body composition in Iranian overweight and obese women: a cross-sectional study.** *BMC Res Notes* 2020, **13:**544.

**Table S1.** The characteristics of demographic, anthropometric and laboratory parameters among study subjects.

| **Variable** | **Mean** ±**SD or Frequency (percentage)** |
| --- | --- |
| **Age (years)** | 8.32±36.52 |
| **Weight (Kg)** | 78.97±10.76 |
| **Height (cm)** | 161.38±5.70 |
| **BMI (Kg/m2)** | 30.33±3.65 |
| **WC (cm)** | 98.45±9.24 |
| **WHR** | 0.93±0.05 |
| **PA (MET/Min/wee)** |  |
| **Low** | 163 (55) |
| **Moderate** | 117 (39) |
| **High** | 10 (5) |
| **CRP (mg/L)** | 4.22±4.61 |
| **IL-1β (mg/L)** | 2.73±0.94 |
| **TGF-β (mg/L)** | 87.98±113.42 |
| **Galectin-3** | 4.09±7.26 |
| **MCP-1** | 78.92±48.88 |
| **Total Energy Intake (kcal)** | 2521.54±737.87 |
| **Total Carbohydrate intake (g)** | 387.50±118.73 (Minimum: 114.57, Maximum: 722.31) |
| **Energy intake from Carbohydrates (%)** | 57.18±4.01 (Minimum: 56.01, Maximum: 75.00) |
| **Total Fat Intake (g)** | 79.18±27.45 (Minimum: 21.15, Maximum: 160.41) |
| **Energy intake from Fats (%)** | 28.00±3.76 (Minimum: 16.26, Maximum: 35.20) |

**Notes: Mean± SD: mean ± standard deviation; BMI: Body mass index; WC: Waist Circumference; WHR: Waist hip ratio; PA: Physical activity, CRP: C-reactive protein; IL1β: Interleukin 1 Beta; TGF-β: Transforming growth factor beta; MCP-1: Monocyte chemoattractant protein-1.**

**Table S2.** The general characteristics and inflammatory parameters of participants across tertiles of dietary carbohydrate intake.

| **Variable** | **T1**  Mean± SD | **T2**  Mean± SD | **T3**  Mean± SD | $\boldsymbol{P-value}$ |
| --- | --- | --- | --- | --- |
| **Age (years)** | 34.97±8.68 | 36.80±7.72 | 37.99±8.28 | **0.01** |
| **Weight (Kg)** | 80.65±10.93 | 77.31±10.99 | 77.61±9.36 | **0.02** |
| **Height (cm)** | 162.32±5.58 | 160.36±5.94 | 160.36±5.65 | **0.01** |
| **BMI (Kg/m^2^)** | 30.65±3.94 | 29.80±3.25 | 30.28±3.50 | 0.26 |
| **WC (cm)** | 99.66±9.66 | 97.09±9.44 | 97.72±8.32 | 0.19 |
| **WHR** | 0.93±0.05 | 0.92±0.05 | 0.92±0.04 | 0.38 |
| **CRP (mg/L)** | 4.34±4.66 | 3.72±4.63 | 4.57±4.58 | 0.49 |
| **IL-1β (mg/L)** | 2.81±1.02 | 2.81±1.02 | 2.62±0.86 | 0.70 |
| **TGF-β (mg/L)** | 62.08±21.04 | 67.04±12.85 | 123.43±182.25 | 0.69 |
| **Galectin-3 (mg/L)** | 6.36±9.34 | 2.53±2.95 | 2.63±5.69 | 0.08 |
| **MCP-1 (mg/L)** | 70.28±26.95 | 74.86±26.10 | 90.54±71.46 | **0.048** |

**One-way analysis of variance. Mean± SD: mean ± standard deviation; BMI: Body mass index; WC: Waist Circumference; WHR: Waist hip ratio; CRP: C-reactive protein; IL1β: Interleukin 1 Beta; TGF-β: Transforming growth factor beta; MCP-1: Monocyte chemoattractant protein-1.**

**Table S3.** The general characteristics and inflammatory parameters of participants across tertiles of dietary fat intake.

| **Variable** | **T1**  Mean± SD | **T2**  Mean± SD | **T3**  Mean± SD | $\boldsymbol{P-value}$ |
| --- | --- | --- | --- | --- |
| **Age (years)** | 35.91±8.23 | 36.67±8.29 | 36.80±8.89 | 0.72 |
| **Weight (Kg)** | 79.93±10.24 | 79.15±10.31 | 77.08±10.83 | 0.14 |
| **Height (cm)** | 162.37±5.69 | 161.16±5.70 | 160.39±5.75 | **0.05** |
| **BMI (Kg/m^2^)** | 30.37±3.45 | 30.51±3.70 | 30.00±3.73 | 0.55 |
| **WC (cm)** | 99.24±8.69 | 98.37±9.42 | 97.44±9.37 | 0.48 |
| **WHR** | 0.93±0.04 | 0.92±0.05 | 0.92±0.05 | 0.63 |
| **CRP (mg/L)** | 4.43±4.36 | 3.70±4.52 | 4.87±5.02 | 0.26 |
| **IL-1β (mg/L)** | 2.62±0.80 | 2.74±0.97 | 2.78±1.03 | 0.87 |
| **TGF-β (mg/L)** | 61.64±19.09 | 213.68±243.36 | 52.95±12.02 | 0.11 |
| **Galectin-3 (mg/L)** | 3.68±6.41 | 5.52±8.79 | 2.37±4.79 | 0.25 |
| **MCP-1 (mg/L)** | 73.93±37.34 | 86.99±61.78 | 70.55±33.50 | 0.14 |

**One-way analysis of variance. Mean± SD: mean ± standard deviation; BMI: Body mass index; WC: Waist Circumference; WHR: Waist hip ratio; CRP: C-reactive protein; IL1β: Interleukin 1 Beta; TGF-β: Transforming growth factor beta; MCP-1: Monocyte chemoattractant protein-1.**
